# Opioid users show worse baseline knee osteoarthritis and faster progression of degenerative changes: a retrospective case-control study based on data from the Osteoarthritis Initiative (OAI)

**DOI:** 10.1186/s13075-021-02524-9

**Published:** 2021-05-22

**Authors:** Jannis Bodden, Gabby B. Joseph, Silvia Schirò, John A. Lynch, Nancy E. Lane, Charles E. McCulloch, Michael C. Nevitt, Thomas M. Link

**Affiliations:** 1 Department of Radiology and Biomedical Imaging, University of California, San Francisco, 185 Berry Street, Lobby 6, Suite 350, San Francisco, CA 94107 USA; 2 Department of Radiology, Klinikum Rechts der Isar, Technical University of Munich, Ismaninger Str. 22, 81675 Munich, Germany; 3 Department of Epidemiology and Biostatistics, University of California, San Francisco, San Francisco, CA USA; 4 Center for Musculoskeletal Health and Department of Medicine, University of California, Davis, Davis, CA USA

**Keywords:** Osteoarthritis, Knee, Magnetic resonance imaging, Opioids, Pain, Osteoarthritis Initiative

## Abstract

**Background:**

Opioids are frequently prescribed for pain control in knee osteoarthritis patients, despite recommendations by current guidelines. Previous studies have investigated the chondrotoxicity of different opioid subtypes. However, the impact opioids may have on progression of osteoarthritis in vivo remains unknown. The aim of this study was thus to describe the associations between opioid use and knee structural changes and clinical outcomes, over 4 years.

**Methods:**

Participants with baseline opioid use (*n*=181) and who continued use for ≥1 year between baseline and 4-year follow-up (*n*=79) were included from the Osteoarthritis Initiative cohort and frequency matched with non-users (controls) (1:2). Whole-Organ Magnetic Resonance Imaging Scores (WORMS) were obtained, including a total summation score (WORMS total, range 0–96) and subscores for cartilage (0–36), menisci (0–24), and bone marrow abnormalities and subchondral cyst-like lesions (0–18, respectively). Knee Injury Osteoarthritis Outcomes score (KOOS) symptoms, quality of life (QOL), and pain were also obtained at baseline and follow-up (range 0–100; lower scores indicate worse outcomes). Using linear regression models, associations between baseline and longitudinal findings were investigated. As pain may modify observations, a sensitivity analysis was performed for longitudinal findings. All analyses were adjusted for sex, BMI, age, race, and Kellgren-Lawrence grade.

**Results:**

Opioid users had greater structural degeneration at baseline (WORMS total: Coef. [95% CI], *P*; 7.1 [5.5, 8.8], <0.001) and a greater increase over 4 years (4.7 [2.9, 6.5], <0.001), compared to controls. Cartilage and meniscus scores increased greater in opioid users, compared to controls (*P*≤0.001), and findings withstood the adjustment for baseline pain (*P*≤0.002). All baseline KOOS scores were lower in opioid users compared to controls (*P*<0.001). QOL loss was greater, when adjusted for baseline KOOS pain (QOL −6.9 [−11.6, −2.1], 0.005).

**Conclusions:**

Opioid users had worse baseline knee structural degeneration and faster progression. Opioid use was also associated with worse symptoms, pain, and QOL. Furthermore, QOL loss was greater in opioid users compared to controls, when adjusted for baseline KOOS pain, indicating that opioids may not be suited to prevent subjective disease progression in KOA patients.

## Introduction

Knee osteoarthritis (KOA) is the most common type of osteoarthritis, with an estimated prevalence of 27% at the age of 70, and with changing demographics, prevalence is increasing [1, 2]. KOA is associated with disability and pain and the latter is frequently treated with pain medication. Although opioids are not superior to non-steroidal anti-inflammatory drugs in pain management in KOA, they are frequently prescribed [3, 4]: DeMik et al. found opioid prescription rates of 15.9% for pain management in KOA in patients between 2007 and 2014. But the problem is not confined to the USA. A study investigating opioid use among KOA patients in Sweden found prescription rates twice as high compared to patients without osteoarthritis [5].

Treatment of KOA symptoms with opioids is not recommended, as opioids do not sufficiently reduce pain or disability, compared to placebo, but are associated with a broader spectrum of common side effects, including nausea, constipation, dizziness, somnolence, and addiction [6, 7]. Particularly, dizziness and somnolence are more common among opioid users and are associated with an increased fall risk [8–11]. Moreover, a higher mortality rate among KOA patients receiving tramadol for pain control has been reported [12–14]. In addition to known systemic adverse effects, chondrotoxicity of meperidine has been shown in cartilage specimens ex vivo [15]. Furthermore, a randomized trial that assessed treatment-related effects of opioids and non-steroidal anti-inflammatory drugs reported an association between the use of transdermal fentanyl and radiographic KOA progression over 12 weeks [15, 16]. However, most previous studies suffer from a low external validity, adherent to in vitro designs, and the aforementioned in-patient trial used radiographs to measure outcomes, which have been shown to be inferior to magnetic resonance (MR) imaging, in particular in early stages of the degenerative disease [17–19].

Thus, the aim of this study was thus to investigate associations between opioid use with MR-based structural joint degeneration outcomes and symptom-oriented outcome measures, cross-sectionally, and over 4 years.

## Methods

### Participant selection

Subjects were obtained from the Osteoarthritis Initiative (OAI, https://oai.nih.gov) database, an observational multi-center study (*n* = 4796 participants) aiming to investigate associations of biomarkers and osteoarthritis (OA). Obtained datasets included the medical drug inventory (MIF), MR imaging datasets of the right knee, and knee radiographs. Moreover, KOOS quality of life (QOL), symptom severity, and pain scores were obtained from the joint pain dataset (JointSx) [20].

**Fig. 1:**
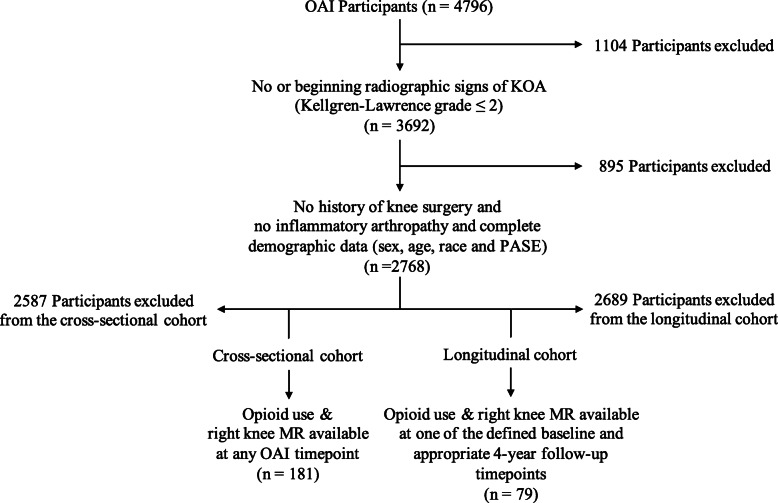
Flow chart depicting inclusion and exclusion criteria for opioid user cohorts. Cross-sectional opioid use was defined by the MIFUSE variable (MIFUSE = 1). Longitudinal opioid use was defined as opioid use at baseline and 4-year follow-up (MIFUSE = 1, respectively) and opioid use duration of at least 1 year at follow-up (MIFDUR ≥ 3)

A flow chart depicting inclusion and exclusion criteria is presented in Fig. 1. Participants in the cross-sectional cohort were included from OAI baseline, 24-month, 48-month, 72-month, and 96-month visits. Participants in the longitudinal cohort were included from three different baselines, with individual 4-year follow-up timepoints: (i) OAI baseline visit to 48-month visit, (ii) 24-month visit to 72-month visit, and (iii) 48-month visit to 96-month visit. Inclusion of participants was limited to one timepoint or one time period, for the cross-sectional and longitudinal cohorts respectively. As a first step to limit the inherent confounding-by-indication bias, eligibility criteria included a Kellgren-Lawrence grade ≤ 2 at the right knee at baseline, for each participant, as knee pain has previously been shown to correlate with the radiographic stage of KOA [21]. Moreover, participants with a history of (i) knee surgery on either side or (ii) history of inflammatory arthropathies were excluded, (i) to avoid the inclusion of patients that used opioids in a perioperative setting and (ii) to exclude KOA progression due to inflammatory arthropathies. Each individual had to have attended the baseline knee MR visit, and participants included in the longitudinal analysis also had to have attended the 4-year follow-up MR visit. Knee Injury Osteoarthritis Outcomes scores (KOOS) for symptom severity and pain and quality of life (QOL) were documented in each subject for individual baseline and follow-up visits, respectively. KOOS scores were chosen over other available scores for their strict focus on knee-related symptoms, quality of life, and pain [20]. Ranging from 0 to 100, a score of 0 reflects the worst possible outcome, while a score of 100 means that no impairment was reported by the participant.

### Definition of opioid use

Opioid users were identified based on medication use as reported in the MIF dataset: Active ingredients of medication were determined using the variable INGCODE, and opioid-containing drugs were identified (opioid (INGCODE): tramadol (28080854); codeine (48000063); propoxyphene (28080840); hydrocodone (48000072); fentanyl (28080810); pentazozine (28080892); methadone (28080818); oxycodone (28080883); hydromorphone (28080813); morphine (28080819); oxymorphone (28080886)). Further, the current use of medication (MIFUSE) and the duration of medication use (MIFDUR) were assessed for each opioid medication, in each participant. Opioids were further divided into “weak” (tramadol, codeine, propoxyphene) and “strong” (hydrocodone, fentanyl, pentazozine, methadone, hydromorphone, morphine, oxymorphone) opioids, according to the WHO and British Pain Society criteria [22, 23].

Participants included in the opioid user cohort of our cross-sectional analysis reported the use of opioid-containing medication within 1 month previous to the baseline visit (MIFUSE = 1). Individuals in the longitudinal opioid user cohort had reported (i) the use of opioid-containing medication at baseline (MIFUSE = 1) and (ii) the use of opioids for the duration of at least 1 year between baseline visit and follow-up (MIFUSE = 1, MIFDUR ≥ 3). Participants included in the control cohort did not report opioid-containing medications at baseline or any time during the 4-year follow-up, as indicated by INGCODE. Combining all three baselines, 181 individual opioid users were included in the cross-sectional analysis. From all three time-spans combined, 79 opioid-using participants met the eligibility criteria to be included in the longitudinal analysis. Controls were frequency matched to opioid users in a two-to-one ratio, using age, sex, race, and the body mass index (BMI) as matching factors [24]. The cross-sectional control cohort consisted of *n* = 362 OAI participants from the OAI baseline, and the longitudinal control cohort of *n* = 158 participants from the time period OAI baseline to 48-month visit.

### Image acquisition and analysis

MR exams were obtained through the OAI and were acquired on four identical 3.0 Tesla MR scanners (Siemens Magnetom Trio, Erlangen, Germany). The scanning protocol of the knee has previously been published and consisted of (i) a coronal 2D intermediate-weighted (IW) turbo spin-echo (TSE) sequence; (ii) a sagittal, fat-saturated (FS) 2D IW TSE sequence; (iii) a coronal 3D fast low angle shot with water excitation (FLASH WE) sequence; and (iv) a sagittal 3D dual-echo steady state sequence with water excitation (DESS WE) [25, 26]. Additional information on imaging protocols used in the OAI is available online (https://oai.epi-ucsf.org/datarelease/operationsManuals/MRI_ManualRev.pdf).

Image analysis was performed on picture archiving communication system workstations (Agfa, Ridgefield Park, NJ, USA). Structural degenerative joint disease was semi-quantitatively graded for each exam using a modified version of the Whole-Organ Magnetic Resonance Imaging Score (WORMS) system in all patients and controls, as previously described [27, 28]. Accordingly, cartilage lesions were graded with scores ranging from 0 (no cartilage signal alteration) to 6 (full-thickness cartilage loss in ≥75% of the region), bone marrow edema-like lesions (BMELs) from 0 (no BMEL) to 3 (BMEL with a max. diameter of ≥ 20 mm), and subchondral cyst-like lesions from 0 (no subchondral cyst) to 3 (subchondral cysts ≥ 5 mm). Scores were graded in six locations (at the patella, trochlea, medial and lateral femoral condyle, medial and lateral tibial plateau, respectively). Meniscal lesions (score range 0 (no meniscal signal alteration) to 4 (maceration)) were also graded in six locations (anterior horn, body, and posterior horn, for medial and lateral meniscus, respectively). WORMS summation scores were calculated for each lesion type (score range: cartilage 0–36, meniscus 0–24, BMEL 0–18, subchondral cyst-like lesions 0–18), for each exam. Moreover, the total WORMS summation score was calculated for each exam by adding WORMS summation scores of each lesion type (cartilage, meniscus, BMEL, and subchondral cyst). Thus, the range of the WORMS total was 0–96. Intra-class correlation coefficients (ICC) demonstrating excellent inter- and intra-reader reproducibility for modified WORMS gradings of cartilage, menisci, and BMEL have previously been reported (ICC_inter-reader_ = 0.97–0.98 and ICC_intra-reader_ = 0.95–0.97, respectively) [28]. In order to avoid multiple testing issues, additional measurements for the degree of structural knee degeneration, such as osteophyte grading or attrition scores, were left out of the analysis.

### Statistical analyses

Statistical analyses were performed using Stata version 15 software (StataCorp, LP, College Station, TX, USA), and analyses were conducted using a statistical significance threshold of *α* = 0.05. Differences in demographics between opioid users and controls were assessed using two-sided independent *t*-tests for continuous variables (BMI, age) and chi-square tests for categorical variables (sex, race). Raw baseline mean outcome values and range were calculated for each outcome, in controls and opioid users, respectively.

The cross-sectional analysis consisted of two parts: First, the associations between opioid use and WORMS/KOOS were assessed using unadjusted linear regression models. Then, the analysis was repeated adjusting for the confounders age, sex, BMI, race, and baseline Kellgren-Lawrence grade.

For the longitudinal analysis, changes in WORMS and KOOS over 4 years were calculated for each individual in the longitudinal cohort. Associations between continued opioid use and change in WORMS/KOOS over 4 years were assessed first, using linear regression models, adjusted for age, sex, BMI, race, and baseline Kellgren-Lawrence grade. Then, a sensitivity analysis was performed to investigate, whether the observed longitudinal associations were explained by structural damage not assessed through Kellgren-Lawrence grade, by adding an additional adjustment for baseline KOOS pain scores to the regression models. The outcome KOOS pain was omitted from the sensitivity analysis, as previous studies have shown that adjusting for baseline values of a variable, when the change of this variable is observed, can lead to problematic interpretations [29]. For this reason, analyses were not adjusted for baseline WORMS scores, either. All regression models were performed by regressing the outcome (e.g., WORMS total) on the opioid status (user vs. non-user) and the adjustment variables listed above. Since the use of opioids was assessed dichotomously, coefficients of regression models indicate the difference in average scores between opioid users and controls (Coefficient = Average_controls_ − Average_opioid users_).

## Results

### Subject characteristics

Subject characteristics of the cross-sectional and longitudinal cohorts are summarized in Table 1. The average age at baseline for opioid users was 60.4 (± SD; ± 9.4) years. Baseline opioid users had an average BMI of 29.6 (± 5.0) kg/m^2^ and 124 out of 181 opioid users (68.5%) were women. Most baseline opioid users were white/Caucasian (*n* (%); 134 (74.0%)), while only one opioid user was Asian (0.6%). The average age of opioid users in the longitudinal cohort was slightly lower compared to the cross-sectional cohort (59.6 ± 8.8 years) and the average BMI was also lower (28.2 ± 4.8). Similar to the cross-sectional cohort, the majority of opioid users in the longitudinal cohort were women (*n* = 53 (67.1%)) and most opioid users were white/Caucasian (60 (75.9%)). Frequency matching of control cohorts to opioid user cohorts was successful for all matched variables (*P* ≥ 0.987, respectively). Means and range for all investigated WORMS and KOOS parameters are listed in Table 1. Notably, the range of WORMS and KOOS was greater in opioid users, compared to controls.

**Table 1:** Cohort characteristics

Parameter		Opioid users		Controls	
Cross-sectional	*n*	181		362	
Age (years; mean, SD)		60.4	9.4	60.4	8.3
BMI (kg/m^2^; mean, SD)		29.6	5.0	29.6	5.0
Sex	Male (*n*, %)	57	31.5	114	31.5
	Female (*n*, %)	124	68.5	248	68.5
Race	Asian (*n*, %)	1	0.6	2	0.6
	White/Caucasian (*n*, %)	134	74.0	268	74.0
	Black/African-American (*n*, %)	46	25.4	92	25.4
Weak opioids^a^ (*n* = 94)	Tramadol (*n*, %)	62	34.3		
	Codeine (*n*, %)	23	12.7		
	Propoxyphene (*n*, %)	9	5.0		
Strong opioids^a^ (*n* = 87)	Hydrocodone (*n*, %)	57	31.5		
	Fentanyl (*n*, %)	1	0.6		
	Methadone (*n*, %)	3	1.7		
	Oxycodone (*n*, %)	22	12.2		
	Hydromorphone (*n*, %)	1	0.6		
	Morphine (*n*, %)	2	1.1		
	Oxymorphone (*n*, %)	1	0.6		
Outcome variables					
WORMS^c^	Total (mean, range)	11.6	(0–48)	18.9	(0–60)
	Cartilage (mean, range)	5.7	(0–20)	8.3	(0–25)
	Meniscus (mean, range)	1.9	(0–13)	3.6	(0–17)
	BMEL (mean, range)	2.1	(0–9)	2.6	(0–12)
	Subchondral cyst (mean, range)	1.0	(0–9)	1.9	(0–10)
KOOS^d^	QOL (mean, range)	73.1	(6.3–100)	60.2	(0–100)
	Symptom (mean, range)	90.0	(46.4–100)	83.4	(32.1–100)
	Pain (mean, range)	88.6	(38.9–100)	78.6	(11.1–100)
Longitudinal	*n*	79		158	
Age (years; mean, SD)		59.6	8.8	59.6	8.7
BMI (kg/m^2^; mean, SD)		28.2	4.8	28.2	4.8
Sex	Male (*n*, %)	26	32.9	52	32.9
	Female (*n*, %)	53	67.1	158	67.1
Race	White/Caucasian (*n*, %)	60	76.0	120	76.0
	Black/African-American (*n*, %)	19	24.1	38	24.1
Weak opioids^a^ (*n* = 38)	Tramadol (*n*, %)	31	39.2		
	Codeine (*n*, %)	4	5.1		
	Propoxyphene (*n*, %)	3	3.8		
Strong opioids^a,b^ (*n* = 41)	Hydrocodone (*n*, %)	31	39.2		
	Fentanyl (*n*, %)	1	1.3		
	Oxycodone (*n*, %)	9	11.4		

^a^According to World Health Organization and British Pain Society criteria [20, 21]. ^b^No participant using methadone, hydromorphone, morphine, or oxymorphone was eligible to be included in the longitudinal cohort. ^c^The maximal possible WORMS total score was 96 and comprised cartilage (score 0–6 in six locations; maximal 36), meniscus (score 0–4, six locations; maximal 24), BMEL (score 0–3, six locations; maximal 18), and subchondral cyst (score 0–3, six locations; maximal 18) scores. A higher WORMS score indicates worse structural knee damage. ^d^KOOS scores range from 0 to 100, with lower KOOS scores reflecting worse reported knee problems. *BMI*, body mass index; *SD*, standard deviation; *WORMS*, Whole-Organ Magnetic Resonance Imaging score; *KOOS*, Knee injury and Osteoarthritis Outcome Score; *BMEL*, bone marrow edema-like lesions; *QOL*, quality of life

Tramadol was the most common weak opioid in the cross-sectional (*n*, %; 62, 34.4%) and longitudinal (31, 39.2%) opioid user cohorts. Participants reporting the use of strong opioids reported the use of hydrocodone (cross-sectional 57, 31.5% and longitudinal 31, 39.2%) and oxycodone (22, 12.2% and 9, 11.4%) most frequently.

### Cross-sectional associations between opioid use and WORMS and KOOS

In the unadjusted analysis, opioid users showed statistically significant, higher scores in baseline WORMS total and all WORMS subscales, compared to controls, respectively (difference in WORMS total [95% CI], *P* value; 7.2 [5.4, 9.1], < 0.001) (Table 2). Moreover, raw differences showed significantly lower QOL, pain, and symptom severity scores in opioid users. Notably, while higher WORMS scores indicate worse structural damage to the knee (e.g., worse cartilage lesions), lower KOOS scores denote worse reported knee problems.

**Table 2:** Differences in baseline scores^a^

	Unadjusted^c^			Adjusted^d^		
Score	Coef.^b^	95% CI	*P*^e^	Coef.^b^	95% CI	*P*^e^
WORMS^f^						
Total	7.2	5.4, 9.1	**< 0.001**	7.1	5.5, 8.8	**< 0.001**
Cartilage	2.6	1.8, 3.5	**< 0.001**	2.6	1.8, 3.4	**< 0.001**
Meniscus	1.9	1.3, 2.4	**< 0.001**	1.9	1.4, 2.4	**< 0.001**
BMEL	0.6	0.2, 0.9	**0.006**	0.5	0.2, 0.9	**0.006**
Subchondral cyst	1.0	0.6, 1.4	**< 0.001**	0.9	0.6, 1.3	**< 0.001**
KOOS^g^						
QOL	−12.9	−16.7, −9.1	**< 0.001**	−12.2	−15.8, −8.5	**< 0.001**
Symptom	−6.6	−8.8, −4.4	**< 0.001**	−6.0	−8.0, −3.8	**< 0.001**
Pain	−10.0	−12.9, −7.1	**< 0.001**	−9.2	−12.1, −6.4	**< 0.001**

^a^Opioid users, *n* = 181; controls, *n* = 362. ^b^Coefficients indicate differences in scores between opioid users and controls. ^c^Unadjusted linear regression models, reflecting raw baseline differences. ^d^Linear regression models, adjusted for age, sex, body mass index, race, and baseline Kellgren-Lawrence grade. ^f^Significant *P* values are printed bold. ^e^A higher WORMS score indicates worse structural knee damage. ^g^KOOS scores range from 0 to 100, with lower KOOS scores reflecting worse reported knee problems. *WORMS*, Whole-Organ Magnetic Resonance Imaging score; *KOOS*, Knee injury and Osteoarthritis Outcome Score; *BMEL*, bone marrow edema-like lesions; *QOL*, quality of life; *Coef.*, coefficient; *95% CI*, 95% confidence interval

**Fig. 2:**
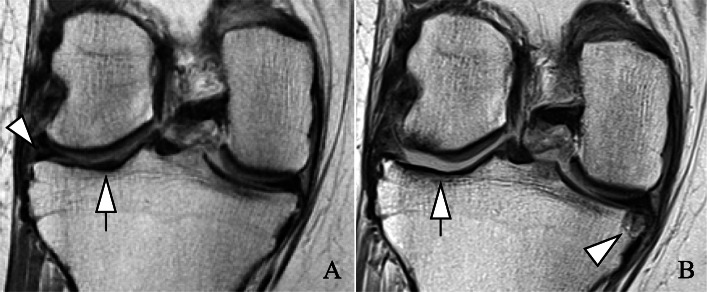
Severe progression of right knee degenerative changes over 4 years in an opioid user. Images were reviewed on picture archiving and communication system (PACS) workstations. Coronal views from intermediate-weighted sequences. **a** Baseline exam. Beginning extrusion and intra-substance lesion of the lateral meniscal body (arrowhead) and cartilage signal abnormality at the lateral tibial plateau (arrow). **b** Deterioration and progressive extrusion of the lateral meniscal body. Full-thickness cartilage loss at the medial femoral condyle (> 1 cm) and medial tibial plateau (> 1 cm) (arrow). Of note, new partial thickness cartilage loss at the medial femoral condyle and new, large subchondral cyst at the medial tibial plateau (arrowhead)

**Fig. 3:**
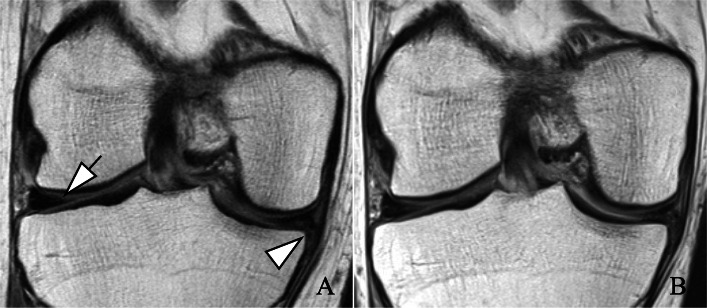
Right knee degenerative changes over 4 years in a control subject. Images were reviewed on picture archiving and communication system (PACS) workstations. Coronal views from intermediate-weighted sequences. **a** Baseline exam. Nondisplaced, horizontal tear in the white zone of the lateral meniscal body (arrow). Complex tear of the medial meniscal body with displaced flap (arrowhead). **b** Four-year follow-up exam. While the horizontal tear of the lateral meniscal body is more distinct, the tear type is stable. Stable complex tear of the medial meniscal body with flap. Of note, absence of cartilage lesions at baseline and follow-up

The adjusted linear regression models also showed statistically significant positive associations between baseline opioid use and greater structural damage to the knee in WORMS total and WORMS subscores (Table 2) (Figs. 2 and 3). Greatest baseline differences in subscores between users and non-users were found for cartilage and meniscal lesions (difference [95% CI], *P* value; cartilage 2.6 [1.8, 3.4], < 0.001; meniscal 1.9 [1.4, 2.4], < 0.001). BMEL and subchondral cyst scores were also greater in opioid users, compared to non-users (BMEL 0.5 [0.2, 0.9], 0.006; subchondral cyst 0.9 [0.6, 1.3] < 0.001). Moreover, opioid users reported statistically significant worse pain, symptom, and QOL scores than controls (pain −9.2 [−12.1, −6.4], < 0.001; symptom −6.0 [−8.0, −3.8], < 0.001; QOL −12.2 [−15.8, −8.5], < 0.001).

### Associations of continued opioid use and progression of WORMS and KOOS

Results of the analysis investigating associations between continued opioid use for at least 1 year during the 4-year follow-up period and WORMS/KOOS are presented in Table 3. Progression of WORMS total scores in opioid users was greater compared to non-users (controls) (difference in progression 4.7 [2.9, 6.5], < 0.001). Opioid users experienced significantly greater score progression compared to controls in WORMS cartilage and meniscus scores, while no statistically significant difference between cohorts was found for BMEL score progression (cartilage 1.5 [0.6, 2.3], 0.001; meniscus 1.7 [1.1, 2.3], < 0.001; BMEL −0.1 [−0.6, 0.3], 0.53). Furthermore, progression of subchondral cyst-like lesions was greater in opioid users compared to controls (0.5 [0.1, 0.9] 0.02). Quality of life loss was also greater in opioid users than in controls, reflected in a greater decrease in KOOS QOL scores over 4 years. However, the difference was not statistically significant (−3.8 [−8.3, 0.7], 0.10). Moreover, no statistically significant difference between cohorts was found in progression of KOOS symptom severity and pain scores (symptom severity 1.3 [−2.1, 4.2], 0.45; pain 0.6 [−3.3, 4.6], 0.75).

**Table 3:** Differences in score progression over 4 years between opioid users^a^ and controls^b^

	Longitudinal analysis			Sensitivity analysis, KOOS pain^e^		
Score	Coef.^c^	95% CI	*P*^d^	Coef.^c^	95% CI	*P*^d^
WORMS^f^						
Total	4.7	2.9, 6.5	**< 0.001**	4.7	2.8, 6.7	**< 0.001**
Cartilage	1.5	0.6, 2.3	**0.001**	1.4	0.5, 2.3	**0.002**
Meniscus	1.7	1.1, 2.3	**< 0.001**	1.7	1.0, 2.3	**< 0.001**
BMEL	−0.1	−0.6, 0.3	0.53	0.1	−0.4, 0.5	0.83
Subchondral cyst	0.5	0.1, 0.9	**0.02**	0.5	0.0, 0.9	**0.03**
KOOS^g^						
QOL	−3.8	−8.3, 0.7	0.10	−6.9	−11.6, −2.1	**0.005**
Symptom	1.3	−2.1, 4.7	0.45	−0.7	−4.4, 3.0	0.72
Pain	0.6	−3.3, 4.5	0.75	Omitted^h^		

^a^*n* = 79. ^b^*n* = 158. ^c^Coefficients indicate differences in scores between opioid users and controls. ^d^Significant *P* values are printed bold. ^e^Results of the sensitivity analysis, adjusted for baseline KOOS pain. ^f^A higher WORMS score indicates worse structural knee damage. ^g^KOOS scores range from 0 to 100, with lower KOOS scores reflecting worse reported knee problems. ^h^KOOS pain was omitted as outcome from the longitudinal analysis. *WORMS*, Whole-Organ Magnetic Resonance Imaging score; *KOOS*, Knee injury and Osteoarthritis Outcome Score; *BMEL*, bone marrow edema-like lesions; *QOL*, quality of life; *Coef.*, coefficient; *95% CI*, 95% confidence interval. All analyses are adjusted for age, sex, body mass index, race, and baseline Kellgren-Lawrence grade

Findings for WORMS total and WORMS subscales remained statistically significant after adjustment for baseline KOOS pain scores in the sensitivity analysis (Table 3). When adjusted for baseline KOOS pain, the models further showed a statistically significant greater loss of QOL. This finding was reflected in a greater score-decrease in opioid users over 4 years, compared to controls (QOL −6.9 [−11.6, −2.1], 0.005). However, the association between opioid use and symptom severity-score progression remained non-significant (*P*=0.72).

## Discussion

This study investigated associations between opioid use in OAI participants and the presence and progression of structural degenerative disease of the knee and KOOS pain, symptom severity, and quality of life as outcome measures. Opioid use was associated with greater baseline degenerative disease. Notably, opioid users also suffered significantly greater pain, worse symptoms, and lower quality of life, indicating insufficient pain control by opioids. Progression of structural degenerative disease over 4 years was greater in opioid users, compared to controls, and findings withstood the adjustments for baseline KOOS pain in the sensitivity analysis. Furthermore, when adjusted for baseline KOOS pain, opioid users demonstrated a significantly greater loss of QOL over 4 years.

Use of opioids in KOA patients is a controversial topic: Although guidelines generally do not recommend opioid use for pain management in osteoarthritis, multiple studies have shown that prescription rates of opioids in large cohorts are still fairly high, at nearly 16% in the USA [4, 5]. In a region-based study in Sweden, the highest prescription rates were found for codeine (43%), followed by oxycodone, tramadol, and morphine (28%, 16%, and 11%, respectively) [5]. Opioid and benzodiazepine prescriptions in outpatient encounters for osteoarthritis in Australia were hydrocodone-acetaminophen (34.3%), oxycodone/oxycodone-acetaminophen (19% and 12.4%, respectively), and tramadol (11.5%) [30]. While the use of tramadol was more frequently documented in this study, rates of strong opioids match the above-mentioned studies.

In this study, we found significantly greater structural damage to the knee at baseline in opioid users. Furthermore, progression of degenerative changes was faster in opioid users compared to controls, particularly for degradation of cartilage and the menisci, and findings withstood the sensitivity analysis. While systemic adverse effects are well known with opioids, effects on the development and progression of osteoarthritis are largely unknown [6]. In a randomized trial with 200 participants, Fujii et al. observed accelerated degenerative disease with loss of radiographic joint space width of more than 50% within 12 weeks in three patients treated with transdermal fentanyl [16]. Further, in vitro effects of opioids on cartilage specimens were investigated by Abrams et al., who reported significant chondrotoxicity of meperidine, while fentanyl and morphine had no effect. Other studies confirmed that neither morphine, nor its metabolite morphine-6-glucuronide, led to significant chondrotoxicity, yet pathways of opioid chondrotoxicity and resulting joint damage remain unknown [31, 32]. But possible effector mechanisms are not limited to direct chondrotoxicity, as previous studies have reported an increased risk of recurrent falls in opioid users, thus also increasing the risk of post-traumatic joint damage and osteoarthritis [8–11]. However, this study does not differentiate between the aforementioned indirect and direct pathways and does not determine causal relationships. Thus, our findings may be mono- or multifactorial, but could also be related to confounding.

Opioid use was further associated with worse baseline pain, symptom severity, and lower QOL, compared to controls without opioid medication. Further, after adjustment for baseline knee pain, greater QOL loss over 4 years underlines that opioids may not provide adequate symptom control in KOA patients. This finding is in line with previous studies that demonstrated associations of opioid use and a reduced health-related QOL in back pain patients, despite the fact that chronic pain conditions themselves have been shown to severely impact QOL [33–35]. Particularly with regard to the known adverse effects and risk of addiction, opioid use for long-term pain management in KOA should be critically questioned [6, 36].

This study has some strengths, including a relatively large and well-characterized longitudinal cohort of individuals with OA or a high risk of OA, and annual imaging and symptom questionnaires, which allow for longitudinal evaluation. However, this study also has limitations: Most importantly, a major confounding-by-indication bias is to be expected in our analysis, meaning that a participant with worse KOA and worse symptoms is thus more likely to use opioids for pain control. Multiple steps were taken to limit this bias: First, as radiographic KOA has been shown to positively correlate with pain, which increases the risk of opioid use, we limited our cohorts to OAI participants with no or mild radiographic KOA and further adjusted for Kellgren-Lawrence grade in the cross-sectional analysis [21]. Second, we further limited the effects of worse baseline KOA on WORMS progression using a sensitivity analysis that added the adjustment for baseline KOOS pain score, as Kellgren-Lawrence grade should not be expected to comprehensively cover all aspects of structural knee degeneration. Nonetheless, the results of this study should not be misinterpreted as implying a true causality of our findings. However, prospective in-patient studies overcoming the confounding-by-indication bias are unlikely to be conducted considering the aforementioned side effects of opioid treatment and the opioid crisis. An alternative way to reduce confounding-by-indication in observational studies could be to investigate KOA development and progression in patients using opioids for other, not KOA-related chronic pain conditions. However, as the OAI questionnaires do not provide information on the indication of opioid treatment, this approach was not feasible for this study. The second limitation of this study concerns the summation of (ordinal) WORMS scores as outcomes. The analyses could be improved by using continuous, truly quantitative data, such as cartilage thickness measurements. However, WORMS and similar semi-quantitative scoring systems are widely used in KOA assessment, as they facilitate to assess a variety of aspects of KOA, such as cartilage lesions, meniscal deterioration, and reactive changes of the subchondral bone [27]. Thus, semi-quantitative WORMS readings performed by experienced and trained radiologists were assessed in this study. Third, the number of opioid users eligible for this study was relatively small, limiting the power of our analyses. However, a comparably low prevalence for opioid use (3.3%) has previously been reported in the OAI progression cohort [37]. Fourth, including BMELs in our analysis imposes a potential source of bias, as BMELs fluctuate over time. However, as BMELs generally tend to increase over time as structural damage to the knee increases and as they correlate well with joint pain, we decided to include BMELs in our analyses [38]. Fifth, we decided to use KOOS scores instead of other, more widely used scores for QOL assessment, e.g., SF-12. However, while SF-12 may be more widely used to assess QOL, the KOOS was designed to specifically address knee-related QOL [20].

## Conclusion

Opioid medication in KOA is associated with worse baseline structural degenerative disease and also with faster progression of degenerative changes. Despite the use of opioids, baseline symptom and pain control were worse in opioid users compared to controls. Loss of QOL was more rapid in opioid users, when adjusted for baseline pain scores, further questioning long-term opioid use, particularly in view of major adverse effects associated with this medication. Strong associations between the continued use of opioids and progression of knee structural degeneration were found that withstood adjustments for baseline Kellgren-Lawrence grade and baseline pain scores. However, findings may in part be related to a confounding bias. Thus, further studies are needed to determine, if opioid use itself imposes a risk for KOA progression.

## Data Availability

The datasets used and/or analyzed during the current study are available in the Osteoarthritis Initiative repository, publicly available at https://nda.nih.gov/oai/ [39].

## Abbreviations

BMEL: Bone marrow edema-like lesion
BMI: Body mass index
Coef.: Coefficient
ICC: Intra-class correlation coefficient
KOA: Knee osteoarthritis
KOOS: Knee Injury Osteoarthritis Outcomes score
MR: Magnetic resonance
OA: Osteoarthritis
OAI: Osteoarthritis Initiative
QOL: Quality of life
WORMS: Whole-Organ Magnetic Resonance Imaging Score
95% CI: 95% confidence interval
